# A Medley of Midbrain Maladies: A Brief Review of Midbrain Anatomy and Syndromology for Radiologists

**DOI:** 10.1155/2012/258524

**Published:** 2012-05-22

**Authors:** Kathleen Ruchalski, Gasser M. Hathout

**Affiliations:** Department of Radiology, David Geffen School of Medicine at UCLA, Los Angeles, CA 90095, USA

## Abstract

The midbrain represents the uppermost portion of the brainstem, containing numerous important nuclei and white matter tracts, most of which are involved in motor control, as well as the auditory and visual pathways. Notable midbrain nuclei include the superior and inferior colliculus nuclei, red nucleus, substantia nigra, oculomotor nuclear complex, and trochlear nucleus. In addition, white matter tracts include the brachium conjunctivum, medial and lateral lemniscus, spinothalamic tracts, and the fiber tracts within the cerebral peduncles. Although neurologically vital, many of these small midbrain nuclei and white matter tracts are not easily individually identified on neuroimaging. However, given their diverse functions, midbrain pathology often leads to distinct clinical syndromes. A review and understanding of the location and relationships between the different midbrain nuclei and fiber tracts will allow more precise correlation of radiologic findings with patient pathology and symptomatology. Particular syndromes associated with midbrain pathology include the Weber, Claude, Benedikt, Nothnagel, and Parinaud syndromes. The oculomotor and trochlear cranial nerves also reside at this level. An understanding of their functions as well as their projected courses from the midbrain towards the eye allows identification of distinct locations which are particularly vulnerable to pathology.

## 1. Introduction

The midbrain is the proximal brainstem bound rostrally by the diencephalon (thalamus and hypothalamus) and adjoined by the pons along its caudal aspect. Because of its close proximity to the cerebral cortex, cerebellum and caudal brainstem, the midbrain contains multiple small fiber tracts which relay vital information amongst these structures. Its role is important in the processing of visual and auditory information, as well as governing motor control. However, many of these tracts as well as midbrain nuclei are not well discriminated by today's neuroimaging techniques. Therefore, the identification of distinct external gross anatomy, including the superior and inferior colliculi and crus cerebri of the midbrain, provides landmarks for evaluation of internal midbrain nuclei, their projections, and their vascular supply. A review of this internal anatomy allows a better understanding of the location of these vital midbrain structures and their functions. Pathology, including infarction and tumor, involving the midbrain, may result in a distinct set of symptoms depending on the portion of midbrain affected. Given the close proximity between these midbrain structures, syndromes may overlap in clinical findings depending on regions of involvement.

Additionally, the oculomotor and trochlear nuclei and cranial nerves originate from this level of the brainstem and extend to the orbit. Evaluation of these cranial nerve projections allows examination of the possible points of pathology and compression that lead to cranial nerve palsies.

It is noted that a review of midbrain anatomy and classical midbrain syndromes may be useful both to clinical neurologists and neuroimagers attempting to correlate imaging findings to clinical signs and symptoms [1].

## 2. Midbrain Anatomy

The interior contents of the midbrain can be anatomically segmented in a dorsal to ventral fashion, including the tectum, tegmentum, and basis [2]. The tectum is the most dorsal aspect of the midbrain and includes the quadrigeminal plate as well as additional gray matter and fiber tracts residing dorsal to the cerebral aqueduct. The centrally located tegmentum represents the largest portion of midbrain real estate and contains cranial nerve nuclei in addition to gray matter and fiber tracts. The remaining ventral midbrain is termed the basis and is comprised of the cerebral peduncles, substantia nigra, crus cerebri, and corticobulbar fibers.

The external midbrain displays distinct landmarks for easy identification. The cerebral peduncles lie along the ventral aspect of the midbrain, just superior to the pons. In addition, the quadrigeminal plate includes the superior and inferior colliculi, which are noted as two paired eminences along the dorsal surface of the midbrain. The oculomotor nerve (cranial nerve III) can be identified along the ventral midbrain within the groove formed between the cerebral peduncles. The trochlear nerve (cranial nerve IV) emerges from the dorsal midbrain near the inferior colliculus.

### 2.1. Level of the Inferior Colliculus

**Figure 1:**
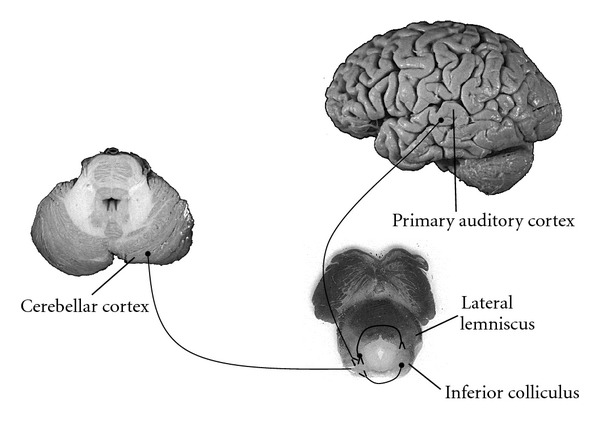
Major afferent connections to the inferior colliculus nucleus. Afferent axons from the medial geniculate, contralateral inferior colliculus, lateral lemniscus, cerebellar cortex and primary auditory cortex connect to the inferior colliculus nuclei. Efferent connections include the medial geniculate body, contralateral inferior colliculus, superior colliculus, lateral lemniscus, and cerebellum (Figure 2).

In the study of neuroanatomy, axial sectioning of the midbrain is commonly performed at the inferior colliculus [2]. The inferior colliculus nuclei represent a triad of gray matter nuclei within the inferior collicular eminences, which play a significant role in the auditory pathway. These three nuclei are layered such that the central nucleus is at the core, surrounded by the pericentral nucleus, and the more laterally placed external nucleus. The central nucleus is the dominant midbrain structure responsible for tonotopically organizing auditory information. The pericentral nucleus and external nucleus both receive auditory and nonauditory inputs to assist in acousticomotor functions, such as guiding auditory attention. Afferent signals are transmitted to the inferior colliculus nuclei via the lateral lemniscus, contralateral inferior colliculus, ipsilateral medial geniculate body, auditory cerebral cortex, and cerebellum (Figure 1).

**Figure 3:**
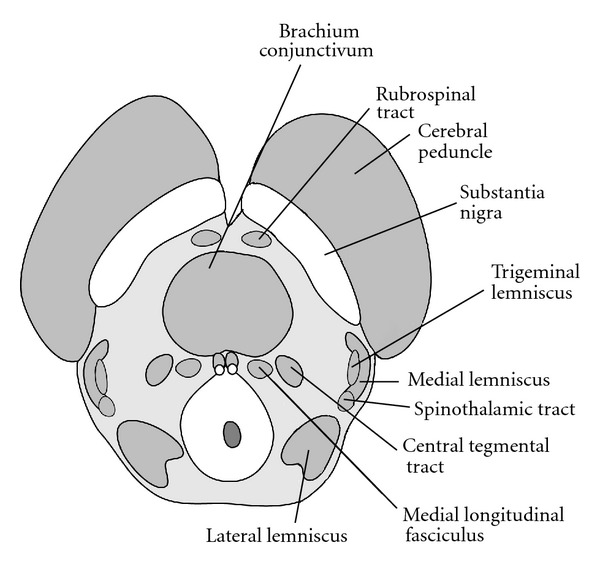
Major white matter tracts within the tegmentum at the level of the inferior colliculus. White matter tracts include ascending sensory fibers within the medial lemniscus, trigeminal lemniscus, and spinothalamic tracts. Decussating cerebellar fibers within the brachium conjunctivum are involved in motor coordination.

The tegmentum contains the majority of the midbrain white matter tracts and nuclear groups at the level of the inferior colliculus [2]. Multiple white matter tracts course through the midbrain with continuations to the cerebral cortex, cerebellum and spinal cord (Figure 3).

The brachium conjunctivum is the largest and most centrally located of these tracts, representing the deccussation of cerebellar axons arising from the superior cerebellar peduncles. These fiber bundles continue to the thalamus and red nucleus and are involved in motor coordination. Sensory fibers course through the lateral tegmentum within the medial lemniscus, trigeminal lemniscus, and spinothalamic tracts. These bundles primarily project to the thalamus. The medial lemniscus relays information regarding proprioception and discriminative touch from the periphery and posterior columns of the spinal cord. Axons from the anterolateral portion of the spinal cord ascend through the spinothalamic tract, conveying sensations of pain and temperature. Facial sensations of pain, temperature, and touch are relayed to the thalamus via the trigeminal lemniscal tract. The medial longitudinal fasciculai are a pair of centrally located fiber tracts, controlling eye movement through connections with the oculomotor (CNIII), trochlear (CN IV) and abducens (CN VI), nerves as well as the vestibulocochlear nerves (CN VIII).

**Figure 4:**
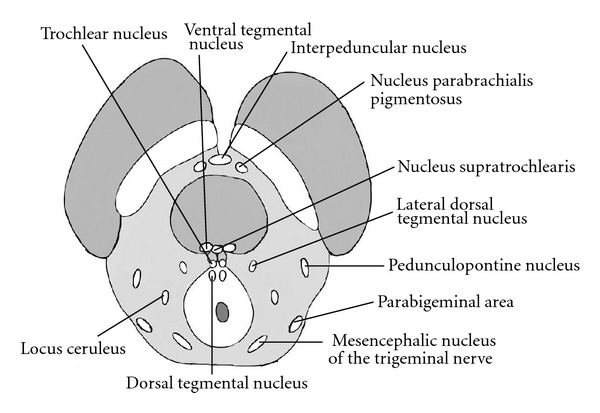
Major nuclear groups within the tegmentum at the level of the inferior colliculus.

Multiple important nuclear groups are found within the tegmentum at this level (Figure 4).

Just dorsal to the substantia nigra, the nucleus parabrachialis pigmentosus is a continuation of the tegemental area of Tsai. The ventral tegmental nucleus resides dorsal to the brachium conjunctivum and receives fibers from the mammillary bodies to assist in regulating emotion and behavior. Within close proximity, the nucleus supratrochlearis (dorsal raphe nucleus) represents the largest serotonergic nucleus within the brain. Projections from the supratrochlearis nucleus extend to the substantia nigra, basal ganglia and cerebral cortex. The dorsal tegmental nucleus is located within the periaqueductal gray matter and sends projections to the reticular formation and autonomic nuclei of the brainstem. The pedunculopontine nucleus, lateral dorsal tegemental nucleus, and parabigeminal areas are cholenergic nuclei within the lateral tegementum. The pedunculopontine nucleus (PPN) receives input from cortical, pallidal, and nigral fibers and is involved in locomotion. The PPN has also been described as a site involved in progressive supranuclear palsy. The parabigeminal areas respond to visual stimuli projecting to and from the superior colliculi. The locus ceruleus is a noradrenergic nucleus with projections throughout the central nervous system, mainly through projections in the central tegmental tract, dorsal longitudinal fasiculus, and the medial forebrain bundle.

**Figure 5:**
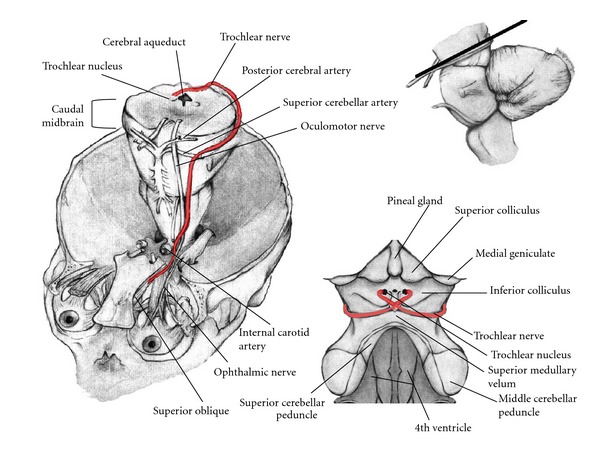
Pathway of the Trochlear Nerve. Elevation and enlargement of the brainstem exposes fibers originating from the trochlear nucleus exiting the dorsal aspect of the midbrain as the Trochlear nerve. This nerve wraps anteriorly and passes between the posterior cerebral artery and superior cerebellar artery along with the oculomotor nerve and continues through the superior orbital fissure to innervate the superior oblique muscle. The dorsal view of the trochlear nerve demonstrates its close proximity to the pineal gland.

The trochlear nucleus may also be identified at the level of the inferior colliculus, residing midline and ventral to the cerebral aqueduct (Figure 5).

Axons from the trochlear nucleus traverse dorsal and lateral to the cerebral aqueduct with the trochlear nerve emerging from the dorsal surface of the midbrain. Externally, the trochlear nerve then courses anteriorly along the surface of the cerebral peduncle. At the ventral surface of the midbrain, these fibers travel next to the oculomotor nerve and together pass between the superior cerebellar and posterior cerebral arteries. The trochlear nerve then continues through the superior orbital fissure to innervate the superior oblique muscle, directing inward torsional gaze.

### 2.2. Level of the Superior Colliculus

**Figure 6:**
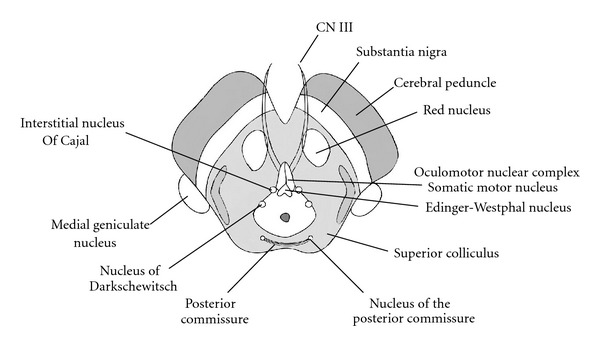
Midbrain anatomy at the level of the Superior Colliculus. The red nucleus and oculomotor nuclear complex are noted at this level in addition to other essential nuclear groups.

The superior colliculus nuclei reside within the tectum as noted externally by the eminences of the superior colliculi (Figure 6).

These nuclei are highly layered structures which govern ocular movements and visual reflexes through afferent connections with the cerebral cortex, retina, spinal cord, inferior colliculus, as well as several additional white matter tracts [2].

The tegmentum is comprised of several nuclear bodies, with the red nucleus and oculomotor complex being the most notable. The red nucleus resides medially within the rostral tegmentum. Little is known about the function of the red nucleus, but its vast afferent pathways are known to include the cerebral and cerebellar cortex. Additionally, efferent projections to the spinal cord, cerebellum, reticular formation, and inferior olive have been noted. Continuations of the previously described white matter tracts within the tegmentum at the level of the inferior colliculus are again seen at this more superior level of the superior colliculus.

The oculomotor nucleus lies ventral of the periaqueductal gray matter within the medial tegmentum. Afferent projections to the oculomotor nucleus originate from the cerebral cortex, cerebellum, mesencephalon, pons, and medulla. The oculomotor nucleus is comprised of a lateral somatic motor column and a medial visceral cell column. The lateral somatic motor column contains three subnuclei: the lateral, medial, and central subnuclei. The lateral subnucleus provides innervation to the ipsilateral inferior rectus, inferior oblique, and medial rectus muscles. The medial subnucleus supplies the contralateral superior rectus muscle. Innervation of the bilateral levator palpebrae superioris is provided by the central subnucleus.

The somatic and visceral fibers from these nuclei coelesce and course ventrally through the midbrain tegmentum and near the red nucleus as well as the inner portion of the cerebral peduncles. Exiting the midbrain within the interpeduncular fossa as the oculomotor nerve, these fibers travel between the superior cerebellar and the posterior cerebral arteries adjacent to the trochlear nerve. Both of these cranial nerves continue to course anteriorly within the subarachnoid space to eventually penetrate the dura into the cavernous sinus. The oculomotor nerve then divides into a superior and inferior division. The superior division innervates the superior rectus and the levator palpebrae superioris muscles, governing upward gaze and lid elevation, respectively. The inferior division provides innervation to the inferior oblique, inferior rectus, and medial rectus muscles. The inferior oblique muscle affects outward torsional gaze. The inferior rectus muscle lowers the eye, while the medial rectus muscle allows adduction.

**Figure 7:**
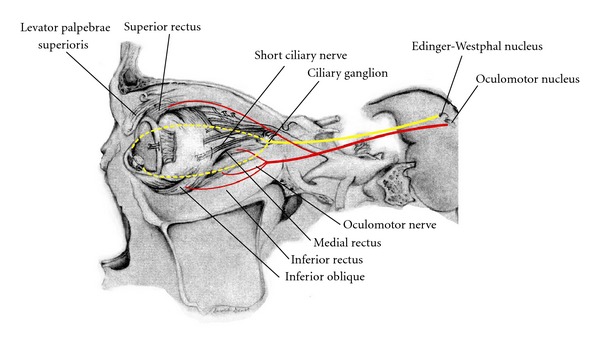
Pathway of the oculomotor nerve. Projections of the somatic and parasympathetic fibers of the oculomotor nerve extend from the midbrain through the cavernous sinus and into the orbit.

The Edinger-Westphal nucleus resides within the visceral cell column and is located dorsal to the somatic oculomotor complex. Preganglionic axons coalesce and travel with oculomotor somatic fibers throughout the pathway to the eye (Figure 7).

Upon entering the orbit, the parasympathetic fibers separate from the nerve to project to the ciliary ganglion. Postsynaptic fibers project to the ciliary body and iris to innervate the sphincter pupillae and ciliaris muscles. The pupillary light reflex allows transmittal of light-activated afferent signals through the optic nerve to project to bilateral Edinger-Westphal nuclei. Therefore, light cast upon one eye results in bilateral and symmetric pupillary constriction. The accommodation reflex directs pupillary constriction, increasing lens curvature and eye convergence with near vision [3].

### 2.3. Ventral Midbrain

**Figure 8:**
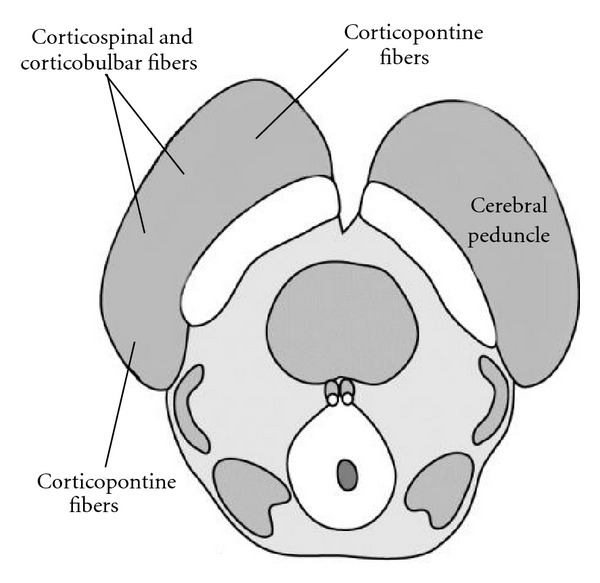
Ventral Midbrain. The ventral midbrain is comprised of the substantia nigra and cerebral peduncles (corticospinal, corticobulbar and corticopontine fiber tracts).

The ventral midbrain appears similar at both the level of the superior and inferior colliculi. Principle structures within this region include the cerebral peduncle and the substantia nigra (Figure 8).

The cerebral peduncles contain corticocerebellar, corticobulbar, and corticospinal fibers. The corticospinal tract occupies roughly the middle three fifths of the cerebral peduncle, with the efferent cortical motor axons arranged somatotopically from medially to laterally as arm, face, and leg. The medial and lateral aspects of the peduncles are composed of corticopontine fibers from the frontal and parieto-occipital regions, respectively. These synapse in the pons, and decussate to enter the contralateral cerebellum through the middle cerebellar peduncles. Corticobulbar fibers, destined for the cranial nerve nuclei, occupy a dorsomedial position in relation to the corticospinal tracts.

**Figure 9:**
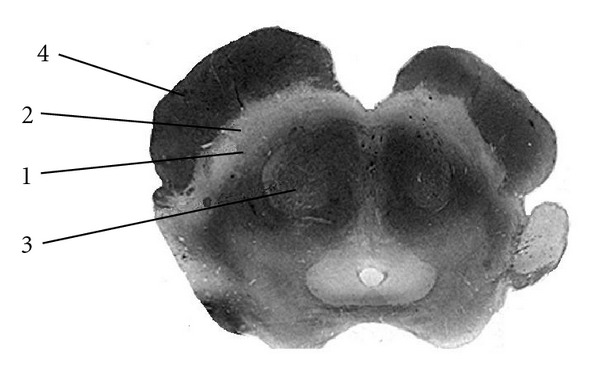
Substantia Nigra. The normal substantia nigra anatomy is shown on a myelin stained section. This pigmented band of tissue dorsal to the cerebral peduncle is divided into two zones. The zona compacta represents the dorsal layer and appears slightly brighter on MRI and sectioning (1). The zona reticulata is the more ventrally located zone which is slightly darker in hue (2). The red nucleus (3) and cerebral peduncle (4) are also identified on these images.

The substantia nigra is identified as a pigmented band of tissue just dorsal to the cerebral peduncle. It is composed of two zones, a dorsal zona compacta and a ventral zona reticulata. The substantia nigra is noted to play a role in several pathways, including motor control and reward. Involvement of the substatia nigra has been described with Parkinson's disease, Huntington's disease, and multisystem atrophy, with each disease showing a characteristic pattern of neuronal loss. Dopaminergic neurons form the center of the zona compacta, for example, are lost in idiopathic Parkinson's disease (Figure 9). 

### 2.4. Midbrain Vascular Supply

**Figure 10:**
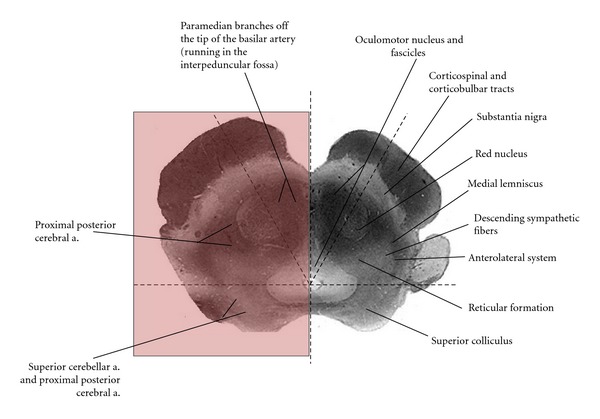
Vascular supply of the midbrain at the level of the superior colliculus is divided into 3 zones. The superior colliculus and tectum are supplied by the superior cerebellar artery. Vascularization of the medial zone is provided by paramedian branches of the basilar artery. The lateral zone is supplied by the posterior cerebral artery.

The midbrain vasculature is primarily supplied by the posterior cerebral circulation, including the basilar, superior cerebellar, and posterior cerebral arteries (Figure 10).

The complexity of this blood supply is demonstrated by variations in vascularity at the inferior colliculus, superior colliculus and pretectal levels. These three levels are further divided axially into medial and lateral zones. The medial zone at each level is supplied by the basilar artery and its paramedian branches. At the level of the inferior colliculus, the lateral zone receives branches from the superior cerebellar artery. The lateral zone at the level of the superior colliculus is supplied by the posterior cerebral artery. However, the superior colliculus and adjacent tectum are vascularized by the superior cerebellar artery. The posterior cerebral artery primarily supplies the lateral zone at the pretectal level [2].

## 3. Cranial Nerve III Palsy

Injury to the oculomotor nerve results in paralysis of the intraocular and extraocular muscles, as well as the levator palpebrae muscle. Damage to the extraocular muscles results in downward and lateral deviation of the eye. Involvement of the intraocular muscles presents with mydriasis as well as divergent gaze. In addition, paralysis of the levator palpebrae muscle causes ptosis [3].

**Figure 11:**
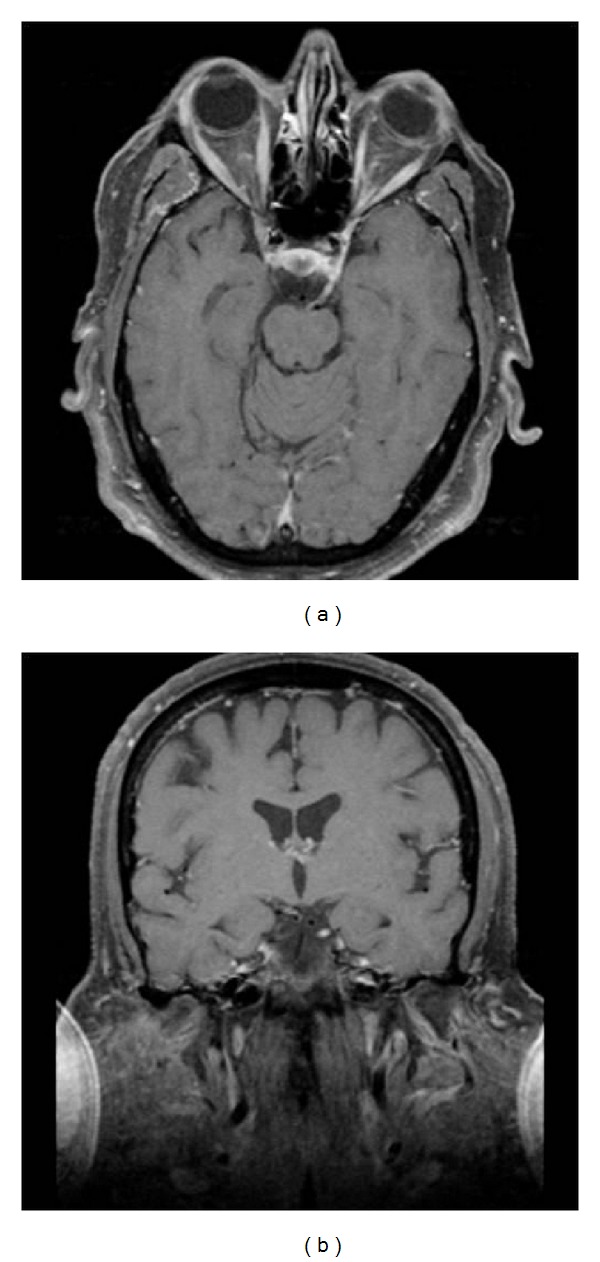
Carcinomatosis involving the left oculomotor nerve. Axial (a) and coronal (b) postcontrast and fat saturated images at the level of the midbrain demonstrate enhancement of the left cranial cranial nerve III. This is compatible with a diagnosis of carcinomatous lymphomatosis.

The oculomotor nerve is susceptible to injury at several points along its course to the orbit. The oculomotor fibers exit the midbrain within the interpeduncular fossa. Displacement of the cerebral peduncle, as seen with temporal lobe herniation, may lead to compression of this nerve. After exiting the interpeduncular fossa, the oculomotor nerve courses between the posterior cerebral artery and superior cerebellar artery. Aneurysmal compression of the nerve at this level may present with isolated cranial nerve III palsy. The oculomotor nerve continues through the cavernous sinus and may suffer from injury at this site when there is cavernous sinus infection, venous thrombosis, or mass lesion. Cavernous sinus pathology, however, typically results in the paralysis of multiple cranial nerves, including the trigeminal (V1 and V2), trochlear (CN IV), and abducens (CN VI) nerves. In addition, carcinomatosis or infectious processes, such as tuberculous meningitis involving the optic chiasm, temporal lobes, or pons, may also affect the oculomotor nerve (Figure 11).

## 4. Midbrain Syndromes

Lesions within the midbrain may result in distinct syndromes, but given the close proximity of these vital nuclei and fiber tracts, these distinct syndromes often result in overlapping symptomatology [1, 2, 4].

### 4.1. Parinaud's Syndrome

**Figure 12:**
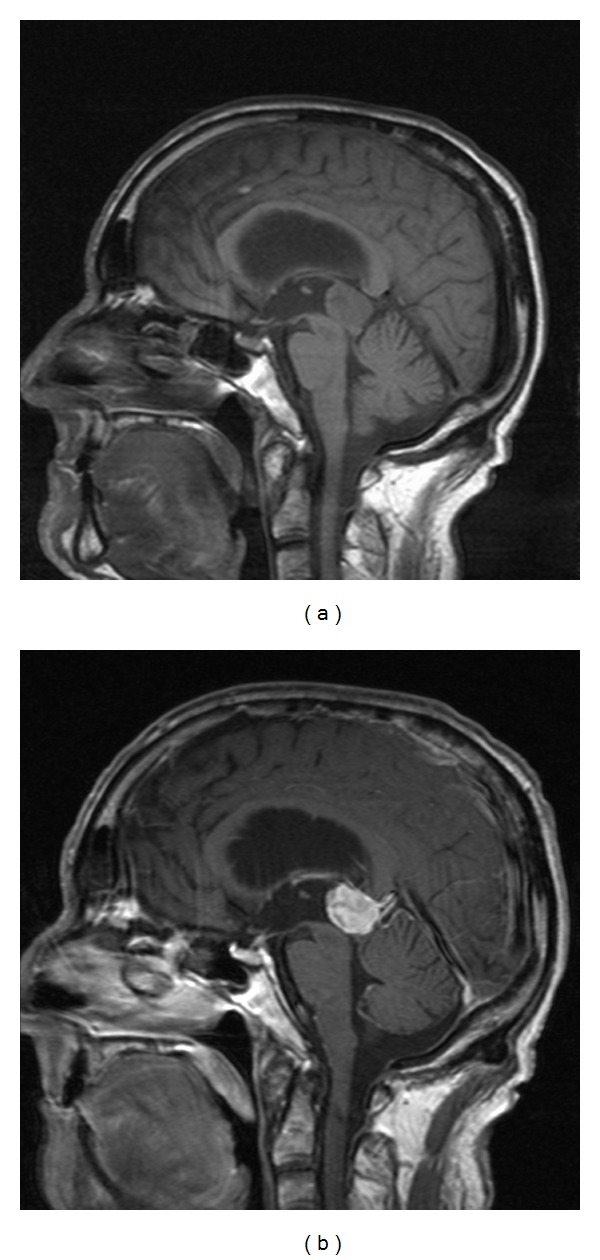
Parinaud's Syndrome. T1 weighted sagittal (a) and postcontrast sagittal (b) images in a 28 year old with a pineocytoma compressing the midbrain tectum. Patient presented with paralysis of upward gaze.

Parinaud's syndrome, also known as the dorsal midbrain syndrome, represents a constellation of symptoms related to compression of the rostral midbrain and pretectum near the level of the superior colliculus, usually due to mass effect from an adjacent pineal tumor [5–7] (Figure 12).

Limited upward gaze is the distinguishing symptom, as the vertical gaze center lies in close vicinity to the superior colliculus, with some of the main nuclei being the interstitial nucleus of Cajal and the rostral interstitial nucleus of the MLF. Interestingly, downward gaze is often preserved. This is in distinction to progressive supranuclear palsy, which also presents with a vertical gaze palsy, but one which preferentially affects downward gaze [8, 9]. The reasons for this difference are not entirely clear, but it has been suggested that the pathways for downward gaze are directly medially out of the rostral interstitial nucleus of the MLF, while those for upward gaze are directed laterally and decussate in the posterior commisure, making them more susceptible to external mass effect. With Parinaud's syndrome, patients may have a downgaze at rest, known as the “setting sun” sign. Patients may also exhibit a pseudo Argyl Robertson pupil, where the pupil is poorly reactive to light but constricts with convergence. This is because the impulses from the optic tract synapse in the pretectal area and then travel via the posterior commisure to both Edinger-Westphal nuclei in the posterior commisure, once again making them susceptible to external compression. Patients may also exhibit lid retraction and convergence-retraction nystagmus.

### 4.2. Weber's Syndrome

**Figure 13:**
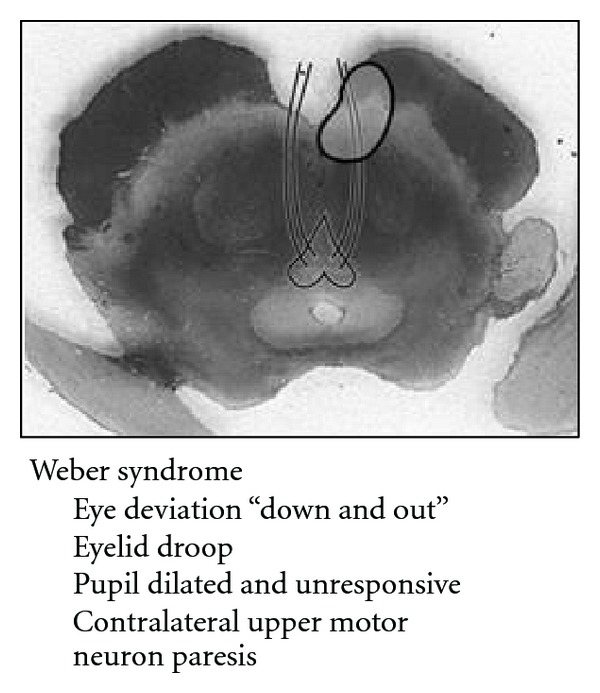
Schematic of Weber's syndrome showing a paramedian midbrain infarct involving the cerebral peduncle and 3rd nerve fascicles.

Infarction of the ventromedial midbrain results in distinct symptoms of Weber Syndrome (Figure 13).

**Figure 14:**
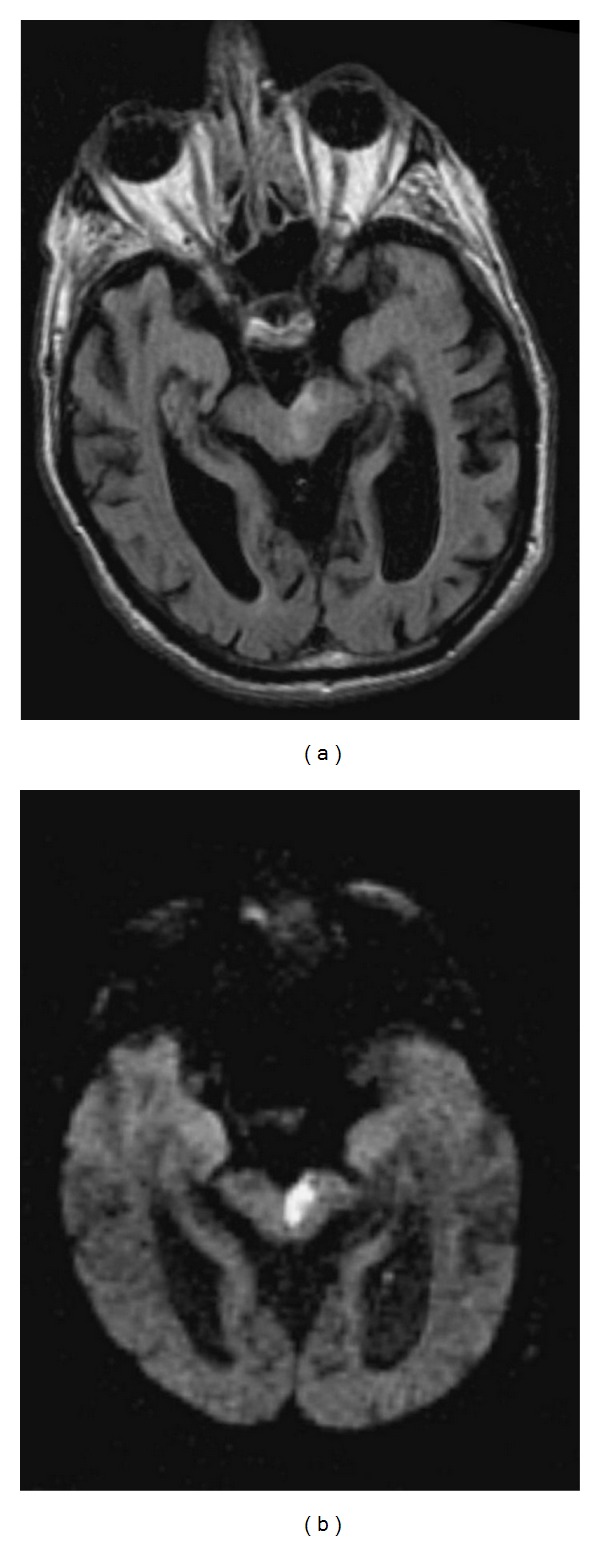
Weber's syndrome. FLAIR (a) and DWI (b) images showing a left paramedian midbrain infarct in a patient with Weber's syndrome. Images used courtesy of Dr. Frank Gaillard at Radiopaedia. org (http://radiopaedia.org/images/94/).

This is characterized predominantly by an ipsilateral third nerve palsy and contralateral weakness [2, 4]. A compromise in the paramedian branches of either the basilar artery or the posterior cerebral artery results in infarction of the oculomotor nucleus and/or fibers, in addition to the cerebral peduncle (Figure 14).

**Figure 15:**
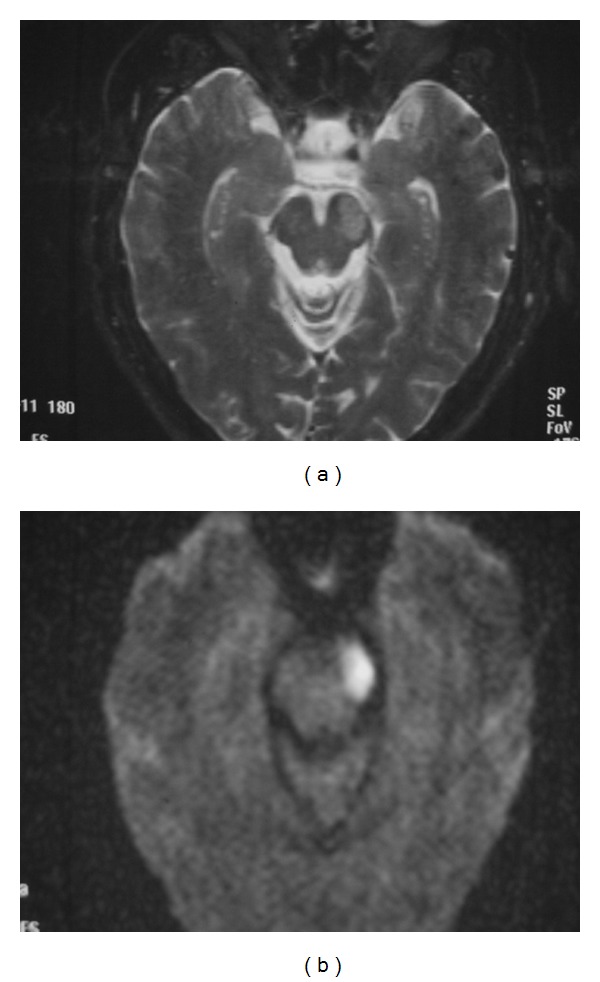
Infarct of the crus cerebri. Axial T2 (a), and DWI (b) MR images of the midbrain with subacute infarction involving the left cerebral peduncle. This patient presented with right-sided face, arm and leg weakness due to involvement of the corticobulbar and corticospinal tracts within the left cerebral peduncle.

Symptoms related to the involvement of cranial nerve III fibers include eye deviation (down and out), diplopia, ptosis, and afferent pupillary defect. Contralateral hemiparesis and lower facial weakness are due to infarction of the crus cerebri, including the corticospinal and corticobulbar tracts respectively. Other possible findings include contralateral parkinsonian rigidity from involvement of the substantia nigra. When the midbrain infarct involves the short circumflex branches and is ventrolateral in the midbrain involving only the cerebral peduncle, there can be sparing of the 3rd nerve (Figure 15).

### 4.3. Claude's Syndrome

**Figure 16:**
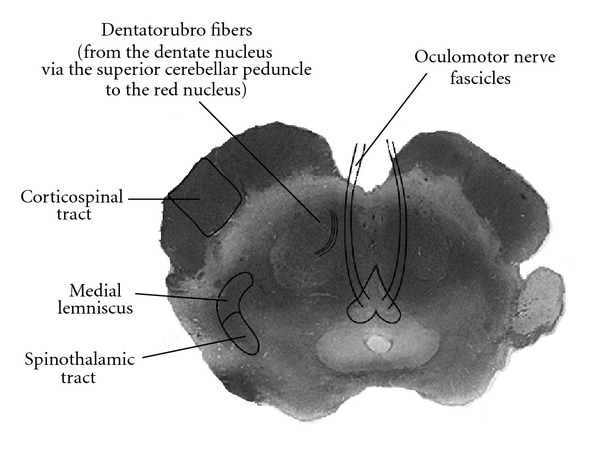
Claude Syndrome. Injury to the dorsal tegmentum, including the oculomotor nerve fascicles, and dentato-rubro fibers.

Claude Syndrome is described as ipsilateral oculomotor nerve palsy with contralateral cerebellar ataxia [2, 10–12] (Figure 16).

**Figure 17:**
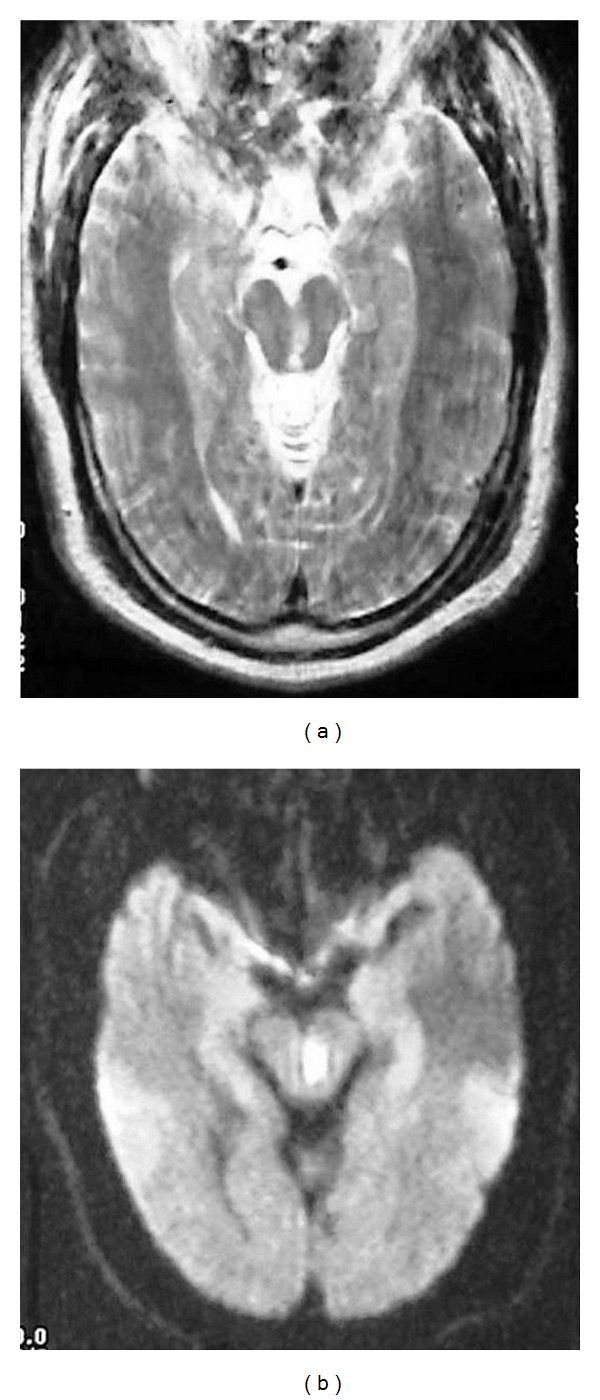
Claude Syndrome. Severely motion degraded axial T2-weighted (a) and DWI (b) images of the midbrain demonstrate a subacute infarct within the left midbrain tegmentum, with extension from the Sylvian aqueduct to the red nucleus. This patient presented with downward abduction of the left eye and right leg incoordination with poor gait. The visual disturbance is related to the involvement of the oculomotor nerve fascicles. Injury to the dentato-rubro fibers and/or red nucleus resulted in ataxia.

Originally characterized as the result of dorsal tegmentum infarction, involvement of the cranial nerve III nucleus and/or nerve fibers leads to oculomotor nerve palsy. Insult to the red nucleus, brachium conjunctivum, or fibers of the superior cerebellar peduncle results in incoordination and cerebellar hemiataxia (Figure 17).

### 4.4. Benedikt's Syndrome

**Figure 18:**
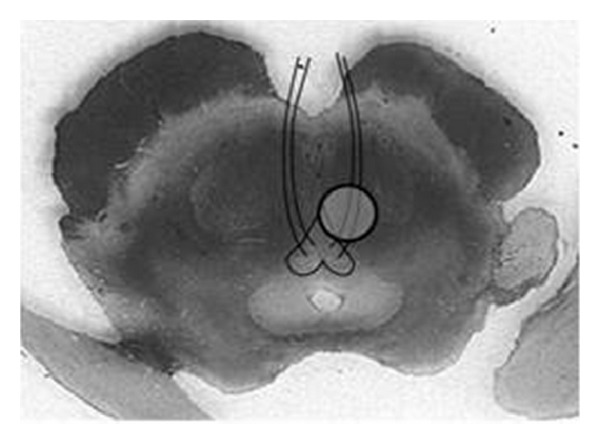
Benedikt syndrome. Localization of oculomotor fascicle injury and symptoms of Benedkit syndrome.

A lesion within the tegmentum of the midbrain may also produce Benedikt Syndrome (Figure 18).

**Figure 19:**
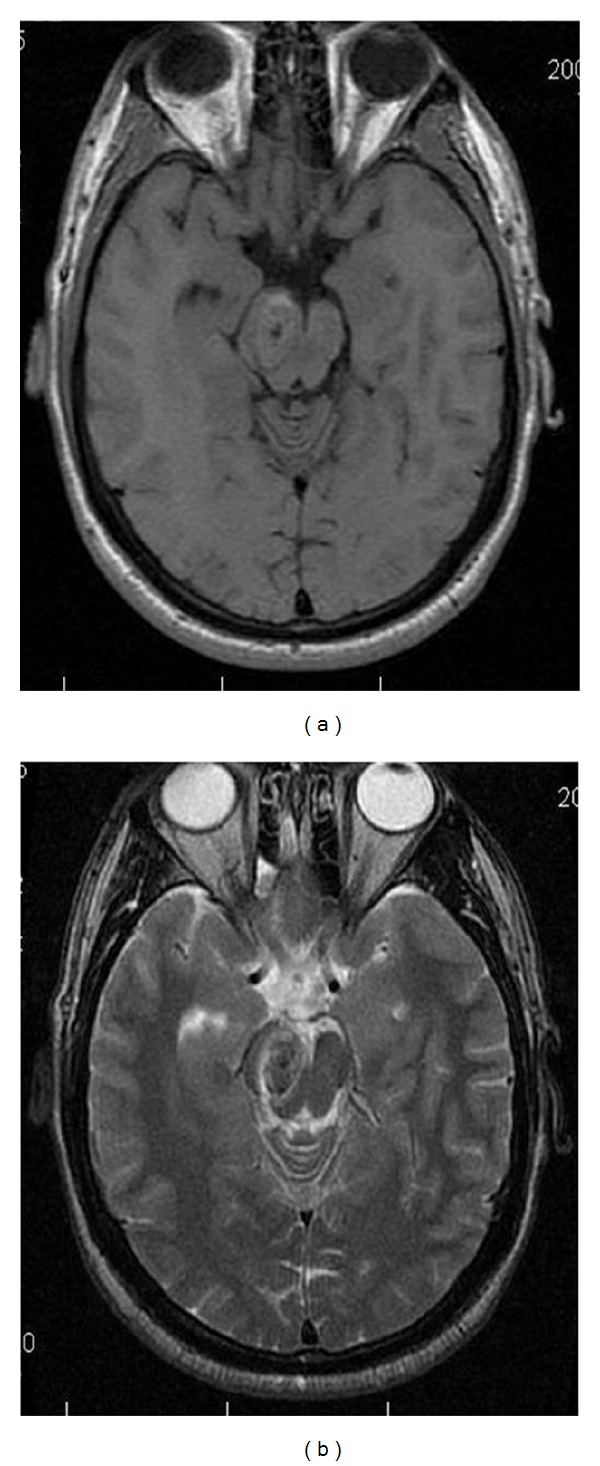
Benedikt Syndome. Ovoid lesion within the midbrain demonstrates isointensity on axial T1-weighted image (a), and slight hypointensity on axial T2-weighted (b) image. These findings are consistent with a cavernous hemangioma within the right midbrain, presenting with symptoms of Benedikt Syndrome.

As with Claude's syndrome, symptoms include incoordination and oculomotor nerve palsy due to involvement of the superior cerebellar peduncle and/or red nucleus, as well as the oculomotor nucleus. In addition, damage to the corticospinal tract resulting in contralateral hemiparesis is the distinct finding in Benedikt's syndrome (Figure 19) [2]. Symptoms are usually a consequence of infarcted branches of the posterior cerebral artery.

### 4.5. Nothnagel's Syndrome

**Figure 20:**
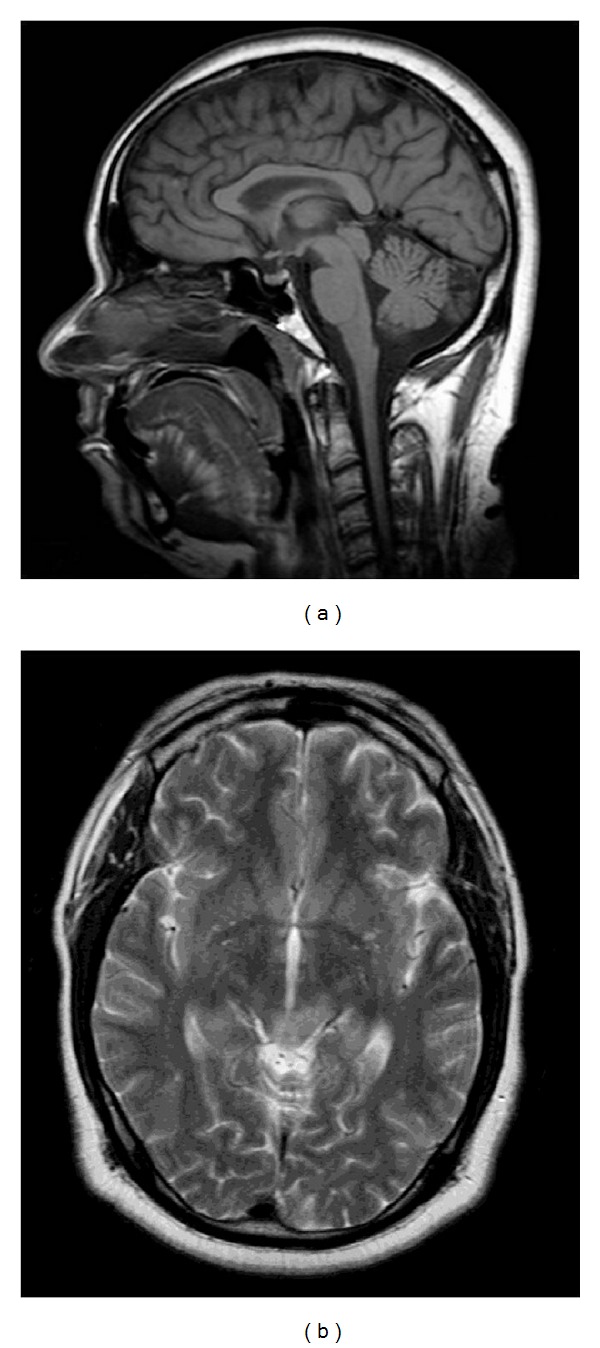
Nothnagel Syndrome. MRI images of the midbrain with enlargement of the midbrain tectum, including the quadrageminal plate, noted on sagittal T1-weighted (a) image. There is associated abnormal T2 hyperintensity seen on accompanying axial T2-weighted (b) image. These findings likely represent tectal glioma with involvement of the oculomotor nuclear complex and decussating fibers of the superior cerebellar peduncle.

Nothnagel's syndrome has been described as unilateral or bilateral oculomotor nerve paralysis and ipsilateral cerebellar ataxia [2, 13]. These symptoms are due to a lesion within the midbrain tectum involving the quadrageminal plate. They result from extension of the lesion to the oculomotor nuclear complex and superior cerebellar peduncles. Pathologically, Nothnagel's syndome has been associated with mass occupying lesions of the midbrain, such as a glioma (Figure 20).

## 5. Conclusion

Although the majority of midbrain nuclei and fiber tracts are not well differentiated on CT or MRI, the use of visible midbrain anatomy may be used to delineate locations of other vital structures. An understanding of the functions and relationships between these different midbrain structures allows for better correlation between regions of pathologic involvement and patient symptomatology.

## Figures and Tables

**Figure 2:**
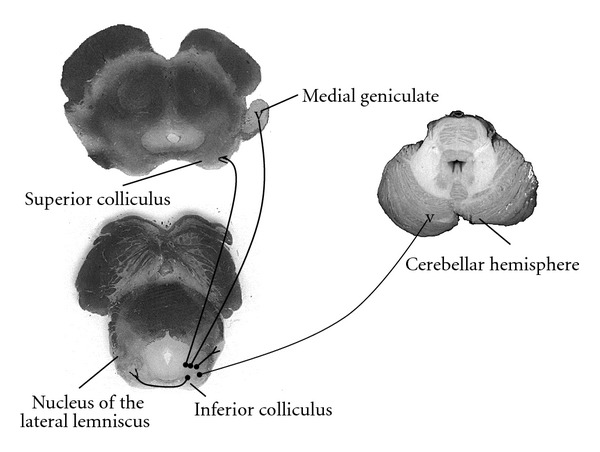
Major efferent connections to the inferior colliculus nuclei. Inferior colliculus nuclei project to the medial geniculate, superior colliculus, cerebellar cortex, contralateral inferior colliculus and lateral lemniscus.
